# Uniformly asymptotic normality of sample quantiles estimator for linearly negative quadrant dependent samples

**DOI:** 10.1186/s13660-018-1788-6

**Published:** 2018-07-28

**Authors:** Xueping Hu, Rong Jiang, Keming Yu, Tong Zhang

**Affiliations:** 1 School of Mathematics and Computation Science, Anqing Normal University, Anqing, P.R. China; 2 Department of Mathematics, Brunel University, Uxbridge, UK; 3 Department of Mathematics, College of Sciences, Donghua University, Shanghai, P.R. China

**Keywords:** 62F12, 62E20, 60F05, Sample quantile, Asymptotic normality, Linearly negative quadrant dependent sequence

## Abstract

In the present article, by utilizing some inequalities for linearly negative quadrant dependent random variables, we discuss the uniformly asymptotic normality of sample quantiles for linearly negative quadrant dependent samples under mild conditions. The rate of uniform asymptotic normality is presented and the rate of convergence is near $O(n^{-1/4} \log n)$ when the third moment is finite, which extends and improves the corresponding results of Yang et al. (J. Inequal. Appl. 2011:83, 2011) and Liu et al. (J. Inequal. Appl. 2014:79, 2014) under negatively associated random samples in some sense.

## Introduction

We first recall the definition of negative (NA, for short), negative quadrant dependent (NQD, for short), and linearly negative quadrant dependent (LNQD, for short) sequences.

### Definition 1.1

(Joag-Dev and Proschan [3])

Random variables $\{X_{i}\}_{1\leq i\leq n}$ are said to be NA if, for every pair of disjoint subsets $A,B\subset \{1,2,\ldots,n\}$,

$$ \operatorname{Cov} \bigl(f(X_{i},i\in A),g(X_{j},j\in B)\bigr) \leq 0, $$

where *f* and *g* are real coordinate-wise nondecreasing functions provided the covariance exists. An infinite sequence of random variables $\{X_{n}\}_{n\geq 1}$ is said to be NA if, for every $n\geq 2$, $X_{1},X_{2},\ldots, X_{n}$ are NA.

### Definition 1.2

(Lehmann [4])

Two random variables *X*, *Y* are said to be NQD if, for any $x,y\in \mathbb{R}$,

$$ P(X< x, Y< y)\leq P(X< x)P(Y< y). $$

A sequence $\{X_{n}\}_{n\geq 1}$ of random variables is said to be pairwise negative quadrant dependent (PNQD, for short) if every pair of random variables in the sequence is NQD.

### Definition 1.3

(Newman [5])

A sequence of random variables $\{X_{i}\}_{1\leq i\leq n}$ is said to be LNQD if, for every pair disjoint subsets $A, B\subset Z^{+}$ and positive $l_{j}'s$, $\sum_{i\in A}l_{i}X_{i}$ and $\sum_{j\in B}l_{j}X_{j}$ are NQD.

### Remark 1.1

It easily follows that if $\{X_{n}\}_{n\geq 1}$ is a sequence of LNQD random variables, then $\{aX_{n}+b \}_{n\geq 1}$ is still a sequence of LNQD, where *a* and *b* are real numbers. Furthermore, NA implies LNQD and PNQD from the above definitions, LNQD random variables are PNQD random variables, but neither reverse is true.

The concept of LNQD sequence was introduced by Newman [5], and subsequently it has been studied by many authors. For instance, Newman investigated the central limit theorem for a strictly stationary LNQD process. Zhang [6] discussed the uniform rates of convergence in the central limit theorem for a LNQD sequence. Wang et al. [7] established the exponential inequalities and complete convergence for a LNQD sequence. Li et al. [8] obtained some inequalities and gave some applications for a nonparametric regression model.

Let $\{X_{n}\}_{n\geq 1}$ be a sequence of random variables defined on $(\Omega,\mathfrak{F}, P)$ with a common marginal distribution function $F(x)=P(X_{1}\leq x)$, where *F* is a right-continuous distribution function. For $p\in (0,1)$, let

$$ \xi_{p}=\inf \bigl\{ x:F(x)\geq p\bigr\} $$

denote the *p*th quantile of *F*, and it is alternately denoted by $F^{-1}(p)$. $F^{-1}(u)$, $0< u<1$, is called the inverse function of *F*. An estimator of the population quantile $F^{-1}(p)$ is given by the sample *p*th quantile

$$ F_{n}^{-1}(p)=\inf \bigl\{ x:F_{n}(x)\geq p\bigr\} , $$

where $F_{n}(x)=\frac{1}{n}\sum_{i=1}^{n}I(X_{i}\leq x)$, $x\in \mathbb{R} $ denotes the empirical distribution function based on the sample $X_{1},X_{2},\ldots,X_{n}$, $n\geq 1$, $I(A)$ denotes the indicator function of a set *A* and $\mathbb{R} $ is the real line.

For a fixed $p\in (0,1)$, denote $\xi_{p}=F^{-1}(p)$, $\xi_{p,n}=F_{n} ^{-1}(p)$ and $\Phi (u)$ represents the distribution function of $N(0,1)$. Liu et al. [2] presented the Berry–Esséen bound of the sample quantiles for a NA sequence as follows.

### Theorem A

*Let* $p\in (0,1)$ *and* $\{X_{n}\}_{n\geq 1}$ *be a second*-*order stationary NA sequence with a common marginal distribution function* *F* *and* $EX_{n}=0$, $n\geq 1$. *Assume that in a neighborhood of* $\xi_{p}$, *F* *possesses a positive continuous density* *f* *and a bounded second derivative* $F''$. *Let* $n_{0}$ *be some positive integer*. *Suppose that there exists* $\varepsilon_{0}>0$ *such that*, *for* $x\in [\xi_{p}-\varepsilon_{0}, \xi_{p}+\varepsilon_{0}]$,

1.1 $$ \bigl\vert \operatorname{Cov}\bigl[I(X_{1}\leq x),I(X_{j}\leq x)\bigr] \bigr\vert \leq C j ^{-5/2},\quad j\geq n_{0}, $$

*and*

1.2 $$ \operatorname{Var}\bigl[I(X_{1}\leq \xi_{p})\bigr]+2\sum _{j=2}^{ \infty }\operatorname{Cov}\bigl[I(X_{1} \leq \xi_{p}),I(X_{j} \leq \xi_{p})\bigr]:= \sigma^{2}(\xi_{p})>0. $$

*Then*

1.3 $$ \sup_{-\infty < x< \infty } \biggl\vert P \biggl( \frac{n^{1/2}(\xi_{p,n}-\xi _{p})}{\sigma (\xi_{p})/f(\xi_{p})}\leq x \biggr) -\Phi (x) \biggr\vert =O\bigl(n ^{-1/6}\log n\bigr),\quad n\rightarrow \infty. $$

For the work on Berry–Esséen bounds of sample quantiles, one can refer to many literature works such as Petrov [9], Shiryaev [10]. The optimal rate is $O(n^{-1/2})$ under the i.i.d. random variables, for the case of martingales, the rate is $O(n^{-1/4}\log n)$ [see [11], Chap. 3]. Recently, Lahiri and Sun [12] obtained the Berry–Esséen bound of the sample quantiles for an *α*-mixing sequence. Yang et al. [1, 13, 14] investigated the Berry–Esséen bound of the sample quantiles for a NA random sequence and a *ϕ*-mixing sequence, respectively, the convergence rate is $O(n^{-1/6}\log n\log \log n)$. Considering other papers about Berry–Esséen bound, Cai and Roussas [15] studied the Berry–Esséen bound for the smooth estimator of a function under association sample. Yang [16–18] investigated uniformly asymptotic normality of the regression weighted estimator for NA, PA, and strong mixing samples, respectively. Liang et al. [19] obtained the Berry–Esséen bound in kernel density estimation for an *α*-mixing censored sample. Under associated samples, Li et al. [20] studied the consistency and uniformly asymptotic normality of wavelet estimator in a regression model.

However, there are very few literature works on uniformly asymptotic normality of sample quantiles for a LNQD sequence which is weaker than a NA sequence. By using some inequalities for LNQD random variables, we investigate the uniformly asymptotic normality of the sample quantiles for a LNQD sequence under mild conditions and obtain the rate of normal approximation, the rate of convergence is near $O(n^{-1/4}\log n)$ provided the third moment is finite, which extends and improves the corresponding results of Liu et al. [2] and Yang et al. [1] in some sense.

The structure of the rest is as follows. In Sect. 2, we give some basic assumptions and the main results. In Sect. 3, proofs of the main results are provided. In the Appendix, some preliminary lemmas are stated. Throughout the paper, $C, C_{1}, C_{2}, \ldots $ denote some positive constants not depending on *n*, which may be different in various places. $\lfloor x\rfloor $ denotes the largest integer not exceeding *x* and second-order stationary means that $(X_{1},X_{1+k}) \stackrel{d}{=}(X_{i},X_{i+k})$, $i\geq 1$, $k\geq 1$.

## Assumptions and main results

In order to formulate our main results, we now list some assumptions as follows.

### Assumption (A1)

(i) $\{X_{n}\}_{n\geq 1}$ is a second-order stationary LNQD sequence with common marginal distribution function *F*. For $p\in (0,1)$, *F* possesses a positive continuous density *f* and a bounded second derivative $F''$ in a neighborhood of $\xi_{p}$.

(ii) $\{X_{n}\}_{n\geq 1}$ is a stationary LNQD sequence with zero mean and finite second moment, $\sup_{j\geq 1}EX_{j}^{2}<\infty $.

### Assumption (A2)

There exists some $\beta >1$ such that

2.1 $$ u(b_{n}):=\sum_{j=b_{n}}^{\infty } \bigl\vert \operatorname{Cov}(X_{1},X_{j}) \bigr\vert =O \bigl(b_{n}^{-\beta }\bigr) $$

for all $0< b_{n}\rightarrow \infty $, as $n\rightarrow \infty $.

### Assumption (A3)

There exist an integer $n_{0}>0$ and some $\varepsilon_{0}>0$ such that, for $x\in [\xi_{p}-\varepsilon_{0},\xi _{p}+\varepsilon_{0}]$,

2.2 $$ \bigl\vert \operatorname{Cov}\bigl[I(X_{1}\leq x),I(X_{j}\leq x)\bigr] \bigr\vert \leq Cj^{-\beta -1},\quad j\geq n_{0},\beta >1. $$

### Assumption (A4)

2.3 $$ \liminf_{n\rightarrow \infty }n^{-1} \operatorname{Var}\Biggl(\sum _{i=1}^{n} X_{i}\Biggr)= \sigma^{2}_{1}>0. $$

### Assumption (A5)

There exist positive integers $p:=p_{n}$ and $q:=q_{n}$ such that, for sufficiently large *n*,

2.4 $$ p+q\leq n,\qquad pq\leq n,\qquad qp^{-1}\leq C< \infty, $$

and let $k:=k_{n}=\lfloor n/(p+q)\rfloor $, as $n\rightarrow \infty $,

2.5 $$ \gamma_{1n}=qp^{-1}\rightarrow 0, \qquad \gamma_{2n}=pn^{-1} \rightarrow 0,\qquad kp/n \rightarrow 1. $$

### Assumption (A6)

There exist an integer $n_{0}>0$ and some $\varepsilon_{0}>0$ such that, for $x\in [\xi_{p}-\varepsilon_{0},\xi _{p}+\varepsilon_{0}]$,

2.6 $$ \bigl\vert \operatorname{Cov}(X_{1},X_{j}) \bigr\vert \leq Cj^{-\beta -1},\quad j\geq n_{0}, \beta >1. $$

### Remark 2.1

Assumptions (A2) and (A5) are used commonly in the literature. For example, Liu [2], Yang [1, 13, 14], Yang [16], Liang [19], and Li [20] used (A5), (A2) and (A4) were used by Liu [2] and Yang [1, 13, 14], (A3) and (A6) were assumed in Liu [2]. Assumption (A5) is easily satisfied, for example, choosing $p=\lfloor n^{2/3}\rfloor $, $q= \lfloor n^{1/3}\rfloor $, $k=\lfloor \frac{n}{p+q}\rfloor =\lfloor n ^{1/3}\rfloor $. It easily follows that $pk/n\rightarrow 1$ implies $qk/n\rightarrow 0$, as $n\rightarrow \infty $.

Our main results are as follows.

### Theorem 2.1

*Suppose that Assumptions* (A1)(ii), (A2), (A4), *and* (A5) *are satisfied*. *If* $|X_{n}|\leq d<\infty $ *for* $n=1,2,\ldots $ , *then*

2.7 $$ \sup_{-\infty < x< \infty } \biggl\vert P \biggl( \frac{\sum_{i=1}^{n} X_{i}}{\sqrt{ \operatorname{Var}(\sum_{i=1}^{n} X_{i})}}\leq x \biggr) - \Phi (x) \biggr\vert \leq C(a_{n}), $$

*where* $a_{n}=(\gamma_{1n}^{1/2}+\gamma_{2n}^{1/2})\log n+\gamma_{2n} ^{(r-2)/2}+n^{-1}+u^{1/3}(q)\rightarrow 0$, *as* $n\rightarrow \infty $, *and* $r>2$.

### Corollary 2.1

*Suppose all the assumptions of Theorem *2.1 *are fulfilled*. *If* $u(n)=O(n^{-3/2})$, $r=3$, *then*

$$ \sup_{-\infty < x< \infty } \biggl\vert P \biggl( \frac{\sum_{i=1}^{n} X_{i}}{\sqrt{ \operatorname{Var}(\sum_{i=1}^{n} X_{i})}}\leq x \biggr) - \Phi (x) \biggr\vert =O\bigl(n^{-1/6}\log n\bigr). $$

### Remark 2.2

We obtain that the rate of normal approximation is $O(n^{-1/6}\log n)$ under a LNQD sequence, which extends the result of Lemma 3.2 in Liu [2] and Lemma 2.1 in Yang [1] in some sense.

### Corollary 2.2

*Suppose all the assumptions of Theorem *2.1 *are satisfied*. *If* $u(n)= [4]O(n^{-3(1-\delta )/2(2\delta -1)})$, $1/2<\delta \leq 2/3$, $r=3 $, *then*

$$ \sup_{-\infty < x< \infty } \biggl\vert P \biggl( \frac{\sum_{i=1}^{n} X_{i}}{\sqrt{ \operatorname{Var}(\sum_{i=1}^{n} X_{i})}}\leq x \biggr) - \Phi (x) \biggr\vert =O\bigl(n^{-(1-\delta )/2}\log n\bigr). $$

### Remark 2.3

The rate of convergence is near $O(n^{-1/4}\log n)$ as $\delta \rightarrow 1/2$ by Corollary 2.2.

### Theorem 2.2

*Let* $\{X_{n}\}_{n\geq 1}$ *be a second*-*order stationary LNQD sequence with a common marginal distribution function* *F* *and* $EX_{n}=0$, $|X_{n}|\leq d<\infty $, $n\geq 1$. *Assumptions* (A5), (A6) *are satisfied and if*

$$ \operatorname{Var}(X_{1})+2\sum_{j=2}^{\infty } \operatorname{Cov}(X_{1},X_{j}):=\sigma^{2}_{0}>0, $$

*then*

2.8 $$ \sup_{-\infty < x< \infty } \biggl\vert P \biggl( \frac{\sum_{i=1}^{n}X _{i}}{\sqrt{n}\sigma_{0}}\leq x \biggr) -\Phi (x) \biggr\vert \leq C(a_{n}), $$

*where* $a_{n}$ *is the same as* (2.7).

Similar to Corollary 2.2, for $r=3$, it follows that the rate of convergence about (2.8) is near $O(n^{-1/4}\log n)$ as $\delta \rightarrow 1/2$.

### Theorem 2.3

*Let Assumptions* (A1)(i), (A3), (A5) *and condition* (1.2) *be satisfied*. *If* $\sup_{n\geq 1}E|X_{n}|^{r}<\infty $ *for some* $r>2$, *then*

2.9 $$ \sup_{-\infty < x< \infty } \biggl\vert P \biggl( \frac{n^{1/2}(\xi_{p,n}-\xi _{p})}{\sigma (\xi_{p})/f(\xi_{p})}\leq x \biggr) -\Phi (x) \biggr\vert \leq C(a_{n}), $$

*where* $a_{n}$ *is the same as* (2.7).

### Remark 2.4

If the third moment is finite, by taking $\beta =3/2$, $p=\lfloor n^{{2}/{3}}\rfloor $, $q=\lfloor n^{1/3}\rfloor $, we obtain that the rate of normal approximation is $O(n^{-1/6}\log n)$ under a LNQD sequence, which extends the result of Theorem 2.1 in Liu [2] and Theorem 1.1 in Yang [1] in some sense.

### Corollary 2.3

*Suppose all the assumptions of Theorem *2.3 *are satisfied*. *If* $u(n)= [4]O(n^{-3(1-\delta )/2(2\delta -1)})$, $1/2<\delta \leq 2/3$, $r=3$, *then*

$$ \sup_{-\infty < x< \infty } \biggl\vert P \biggl( \frac{n^{1/2}(\xi_{p,n}-\xi _{p})}{\sigma (\xi_{p})/f(\xi_{p})}\leq x \biggr) -\Phi (x) \biggr\vert =O\bigl(n ^{-(1-\delta )/2}\log n\bigr). $$

### Remark 2.5

When the third moment is finite, the rate of convergence is near $O(n^{-1/4}\log n)$ as $\delta \rightarrow 1/2$ by Corollary 2.3, when $\delta =2/3$, the rate of convergence is near $O(n^{-1/6}\log n)$.

## Proof of the main results

### Proof of Theorem 2.1

We employ Bernstein’s big-block and small-block procedure and partition the set $\{1,2,\ldots, n\}$ into $2k_{n}+1$ subsets with large block of size $p=p_{n}$ and small block of size $q=q_{n}$, and let $k=k_{n}:=\lfloor \frac{n}{p_{n}+q_{n}} \rfloor $. Define $Z_{n,i}=X_{i}/\sqrt{ \operatorname{Var}(\sum_{i=1}^{n} X_{i})}$, then $S_{n}$ may be split as

$$ S_{n}:=\frac{\sum_{i=1}^{n} X_{i}}{\sqrt{ \operatorname{Var}(\sum_{i=1}^{n} X_{i})}}=\sum_{i=1}^{n} Z _{n,i}=S_{n1}+S_{n2}+S_{n3}, $$

where $S_{n1}=\sum_{j=1}^{k} \eta_{j}$, $S_{n2}=\sum_{j=1}^{k} \xi_{j}$, $S _{n3}= \zeta_{k}$, and $\eta_{j}=\sum_{i=k_{j}}^{k_{j}+p-1} Z_{n,i}$, $\xi_{j}=\sum_{i=l_{j}}^{l_{j}+q-1} Z_{n,i}$, $\zeta_{k}=\sum_{i=k(p+q)+1} ^{n} Z_{n,i}$, $k_{j}=(j-1)(p+q)+1$, $l_{j}=(j-1)(p+q)+p+1$, $j=1,2,\ldots,k$.

By Lemma A.1 with $a=\varepsilon_{1}+\varepsilon_{2}$, we have

3.1 $$ \begin{aligned}[b] \sup_{-\infty < t< \infty } \bigl\vert P(S_{n} \leq t)-\Phi (t) \bigr\vert &=\sup_{-\infty < t< \infty } \bigl\vert P(S_{n1}+S_{n2}+S_{n3}\leq t)-\Phi (t) \bigr\vert \\ &\leq \sup_{-\infty < t< \infty } \bigl\vert P(S_{n1}\leq t)-\Phi (t) \bigr\vert +\frac{a}{\sqrt{2 \pi }} \\ &\quad {}+P\bigl( \vert S_{n2} \vert \geq \varepsilon_{1} \bigr)+P\bigl( \vert S_{n3} \vert \geq \varepsilon _{2} \bigr). \end{aligned} $$

Firstly, we estimate $E(S_{n2})^{2}$ and $E(S_{n3})^{2}$. By the condition $|X_{i}|\leq d$ and Assumption (A4), it is easy to find that $|Z_{n,i}|\leq C_{1}n^{-1/2}$, $|\xi _{j}|\leq C_{1}qn^{-1/2}$, $E\xi _{j}^{2}\leq C_{2}qn^{-1}$, $j=1,2,\ldots,n$. Combining the definition of LNQD with the definition of $\xi_{j}$, $j=1,2,\ldots,k$, we can easily prove that $\{\xi_{i}\} _{1\leq i\leq k}$ is LNQD. It follows from Lemma A.2 that

3.2 $$\begin{aligned}& E(S_{n2})^{2}=E\Biggl(\sum_{j=1}^{k} \sum_{i=l_{j}}^{l_{j}+q-1}Z_{n,i} \Biggr)^{2} \leq C_{2}kq/n=C_{2}qp^{-1}=C_{2} \gamma_{1n}, \end{aligned}$$

3.3 $$\begin{aligned}& E(S_{n3})^{2}=E\Biggl(\sum_{i={k(p+q)+1}}^{n}Z_{n,i} \Biggr)^{2}\leq C_{3}\bigl[n-k(p+q)\bigr]/n \leq C_{3}(p+q)/n=C_{3}\gamma_{2n}. \end{aligned}$$

By (3.2), (3.3) and Lemma A.3, choosing $\varepsilon_{1}=M\gamma_{1n} ^{1/2}(\log n)$, $\varepsilon_{2}= M\gamma_{2n}^{1/2}(\log n)$ and noting that $pq\leq n$, for large enough *M*, *n*, we have

3.4 $$\begin{aligned}& \begin{aligned}[b] P\bigl( \vert S_{n2} \vert > \varepsilon_{1}\bigr) &\leq 2\exp \biggl\{ -\frac{M^{2} \gamma _{1n}\log^{2} n}{2(2 C_{2} \gamma_{1n}+ C_{1} M q/\sqrt{n}\gamma_{1n}^{1/2}\log n)} \biggr\} \\ &\leq 2\exp \biggl\{ -\frac{M^{2}\log^{2} n}{2(2 C_{2}+C_{1} M\log n )} \biggr\} \leq Cn^{-1}, \end{aligned} \end{aligned}$$

3.5 $$\begin{aligned}& \begin{aligned}[b] P\bigl( \vert S_{n3} \vert > \varepsilon_{2}\bigr) &\leq 2\exp \biggl\{ -\frac{M^{2}\gamma _{2n}\log^{2} n}{2(2C_{3} \gamma_{2n}+ C_{1} Mn^{-1/2}\gamma_{2n}^{1/2}\log n)} \biggr\} \\ & \leq 2\exp \biggl\{ -\frac{M^{2}\log^{2} n}{2(2C_{3}+ C_{1} M )} \biggr\} \leq Cn^{-1}. \end{aligned} \end{aligned}$$

Secondly, we estimate $\sup_{-\infty < t<\infty }|P(S_{n1} \leq t)-\Phi (t)|$. Define

$$ s_{n}^{2}:=\sum_{j=1}^{k} \operatorname{Var}(\eta_{j}),\qquad \Gamma_{n}:=\sum _{1\leq i< j\leq k} \operatorname{Cov}(\eta_{i},\eta_{j}). $$

Clearly $s_{n}^{2}=E(S_{n1})^{2}-2\Gamma_{n}$, and since $ES_{n}^{2}=1$, by (3.2) and (3.3) we get that

3.6 $$ \bigl\vert E(S_{n1})^{2}-1 \bigr\vert = \bigl\vert E(S_{n2}+S_{n3})^{2}-2E\bigl[S_{n}(S_{n2}+S_{n3}) \bigr] \bigr\vert \leq C\bigl(\gamma_{1n}^{1/2}+ \gamma_{2n}^{1/2}\bigr). $$

On the other hand, by Assumptions (A1)(ii), (A4), and (A5),

3.7 $$ \begin{aligned}[b] \Gamma_{n} &=\sum_{1\leq i< j\leq k} \sum_{s=k_{i}}^{k_{i}+p-1} \sum _{t=k_{j}}^{k_{j}+p-1}\operatorname{Cov}(Z_{n,s},Z _{n,t}) \\ &\leq C n^{-1}\sum_{i=1}^{k-1}\sum _{s=k_{i}} ^{k_{i}+p-1}\sum _{j=q}^{\infty } \bigl\vert \operatorname{Cov}(X_{1},X _{j}) \bigr\vert \\ &\leq C\bigl[kpu(q)\bigr]/n\leq Cu(q). \end{aligned} $$

From (3.6) and (3.7), it follows that

3.8 $$ \bigl\vert s_{n}^{2}-1 \bigr\vert \leq C\bigl[ \gamma_{1n}^{1/2}+\gamma_{2n}^{1/2}+u(q) \bigr]. $$

We assume that $\eta '_{j}$ are the independent random variables and $\eta '_{j}$ have the same distribution as $\eta_{j}$, $j=1,2,\ldots,k$. Let $H_{n}:=\sum_{j=1}^{k}\eta '_{j}$. It is easily seen that

$$ \begin{aligned}[b] &\sup_{-\infty < t< \infty } \bigl\vert P(S_{n1} \leq t)-\Phi (t) \bigr\vert \\ &\quad \leq \sup_{-\infty < t< \infty } \bigl\vert P(S_{n1}\leq t)-P(H_{n}\leq t) \bigr\vert \\ &\qquad {}+ \sup_{-\infty < t< \infty } \bigl\vert P(H_{n}\leq t)-\Phi (t/s_{n}) \bigr\vert + \sup_{-\infty < t< \infty } \bigl\vert \Phi (t/s_{n})-\Phi (t) \bigr\vert \\ &\quad :=D_{1}+D _{2}+D_{3}. \end{aligned} $$

Let $\phi (t)$ and $\varphi (t)$ be the characteristic functions of $S_{n1}$ and $H_{n}$, respectively. Thus, applying the Esséen inequality(see [9],Theorem 5.3), for any $T>0$,

$$ \begin{aligned} D_{1}&\leq \int_{-T}^{T} \biggl\vert \frac{\phi (t)-\varphi (t)}{t} \biggr\vert \,\mathrm{d}t\\ &\quad {}+T\sup_{-\infty < t< \infty } \int_{ \vert u \vert \leq C/T} \bigl\vert P(H _{n}\leq u+t)-P(H_{n}\leq t) \bigr\vert \,\mathrm{d}u\\ &:=D_{1n}+D_{2n}. \end{aligned} $$

By Assumption (A1)(ii) and Lemma A.4, we have that

$$ \begin{aligned}[b] \bigl\vert \phi (t)-\varphi (t) \bigr\vert &= \Biggl\vert E\exp \Biggl( \mathrm{i}t\sum_{j=1}^{k} \eta_{j} \Biggr) -\prod_{j=1}^{k} E\exp {(\mathrm{i}t\eta_{j})} \Biggr\vert \\ &\leq 4t^{2}\sum_{1\leq i< j\leq k}\sum _{s=k_{i}}^{k _{i}+p-1}\sum_{t=k_{j}}^{k_{j}+p-1} \bigl\vert \operatorname{Cov}(Z_{n,s},Z_{n,t}) \bigr\vert \\ &\leq 4Ct^{2}k pn^{-1}\sum_{j=q}^{\infty } \bigl\vert \operatorname{Cov}(X_{1},X_{j}) \bigr\vert \leq Ct^{2}u(q). \end{aligned} $$

Therefore

3.9 $$ D_{1n}= \int_{-T}^{T} \biggl\vert \frac{\phi (t)-\varphi (t)}{t} \biggr\vert \,\mathrm{d}t\leq Cu(q)T^{2}. $$

It follows from the Berry–Esséen inequality [[9], Theorem 5.7] and Lemma A.2, for $r>2$,

3.10 $$ \begin{aligned}[b] \sup_{-\infty < t< \infty } \bigl\vert P(H_{n}/s_{n}\leq t)-\Phi (t) \bigr\vert & \leq \frac{C}{s_{n}^{r}}\sum_{j=1}^{k}E \bigl\vert \eta '_{j} \bigr\vert ^{r}= \frac{C}{s _{n}^{r}}\sum_{j=1}^{k}E \vert \eta_{j} \vert ^{r} \\ &\leq \frac{Ck[(p/n)]^{r/2}}{s _{n}^{r}}\leq C\frac{\gamma_{2n}^{(r-2)/2}}{s_{n}^{r}}. \end{aligned} $$

Notice that $s_{n}\rightarrow 1$, as $n\rightarrow \infty $ by (3.8). From (3.10), we get that

3.11 $$ \sup_{-\infty < t< \infty } \bigl\vert P(H_{n}/s_{n}\leq t)-\Phi (t) \bigr\vert \leq C \gamma_{2n}^{(r-2)/2}, $$

which implies that

3.12 $$ \begin{aligned}[b] &\sup_{-\infty < t< \infty } \bigl\vert P(H_{n} \leq t+u)-P(H_{n}\leq t) \bigr\vert \\ &\quad \leq \sup_{-\infty < t< \infty } \biggl\vert P \biggl( \frac{H_{n}}{s_{n}} \leq \frac{t+u}{s_{n}} \biggr) -\Phi \biggl( \frac{t+u}{s_{n}} \biggr) \biggr\vert \\ &\qquad {}+\sup_{-\infty < t< \infty } \biggl\vert P \biggl( \frac{H_{n}}{s_{n}} \leq \frac{t}{s_{n}} \biggr) -\Phi \biggl( \frac{t}{s_{n}} \biggr) \biggr\vert + \sup_{-\infty < t< \infty } \biggl\vert \Phi \biggl( \frac{t+u}{s_{n}} \biggr) - \Phi \biggl( \frac{t}{s_{n}} \biggr) \biggr\vert \\ &\quad \leq 2\sup_{-\infty < t< \infty } \biggl\vert P \biggl( \frac{H_{n}}{s_{n}} \leq t \biggr) - \Phi (t) \biggr\vert +\sup_{-\infty < t< \infty } \biggl\vert \Phi \biggl( \frac{t+u}{s _{n}} \biggr) -\Phi \biggl( \frac{t}{s_{n}} \biggr) \biggr\vert \\ &\quad \leq C \biggl( \gamma _{2n}^{(r-2)/2}+ \biggl\vert \frac{u}{s_{n}} \biggr\vert \biggr). \end{aligned} $$

By (3.12), we obtain

3.13 $$ D_{2n}=T\sup_{-\infty < t< \infty } \int_{ \vert u \vert \leq C/T} \bigl\vert P(H _{n}\leq t+u)-P(H_{n}\leq t) \bigr\vert \,du\leq C\bigl(\gamma_{2n}^{(r-2)/2}+1/T \bigr). $$

Combining (3.9) with (3.13) and choosing $T=u^{-1/3}(q)$, we can easily see that

3.14 $$ D_{1}\leq C\bigl(u^{1/3}(q)\bigr)+\gamma_{2n}^{(r-2)/2}), $$

and by (3.11),

3.15 $$ D_{2} =\sup_{-\infty < t< \infty } \biggl\vert P \biggl( \frac{H_{n}}{s _{n}}\leq \frac{t}{s_{n}} \biggr) -\Phi \biggl(\frac{t}{s_{n}} \biggr) \biggr\vert \leq C \gamma_{2n}^{(r-2)/2}. $$

On the other hand, from (3.8) and Lemma 5.2 in [9], it follows that

3.16 $$ \begin{aligned}[b] D_{3}&\leq (2\pi e)^{-1/2}(s_{n}-1)I(s_{n} \geq 1)+(2\pi e)^{-1/2}\bigl(s _{n}^{-1}-1 \bigr)I(0< s_{n}< 1) \\ &\leq C \bigl\vert s^{2}_{n}-1 \bigr\vert \leq C\bigl[ \gamma_{1n} ^{1/2}+\gamma_{2n}^{1/2}+u(q) \bigr]. \end{aligned} $$

Consequently, combining (3.14), (3.15) with (3.16), we can get

3.17 $$ \sup_{-\infty < t< \infty } \bigl\vert P(S_{n1}\leq t)-\Phi (t) \bigr\vert \leq C\bigl[ \gamma_{1n}^{1/2}+ \gamma_{2n}^{1/2}+\gamma_{2n}^{(r-2)/2}+u^{1/3}(q) \bigr]. $$

Finally, by (3.1), (3.4), (3.5), and (3.17), (2.7) is verified. □

### Proof of Corollary 2.1

We obtain it by choosing $p=\lfloor n ^{{2}/{3}}\rfloor$, $q=\lfloor n^{1/3}\rfloor $ in Theorem 2.1. □

### Proof of Corollary 2.2

We obtain it by choosing $p=\lfloor n ^{\delta }\rfloor$, $q=\lfloor n^{2\delta -1}\rfloor $ in Theorem 2.1. □

### Proof of Theorem 2.2

Define $\sigma_{n}^{2}:= \operatorname{Var}(\sum_{i=1}^{n} X_{i})$ and $\gamma (k)= \operatorname{Cov}(X_{i},X_{i+k})$ for $i=1,2,\ldots $ , according to (2.6), for some $\beta >1$, it is checked that

3.18 $$ \sum_{j=b_{n}}^{\infty } \bigl\vert \operatorname{Cov}(X_{1},X_{j+1}) \bigr\vert \leq C\sum _{j=b_{n}}^{\infty }j^{-\beta -1}=O\bigl(b_{n}^{-\beta } \bigr), $$

therefore Assumption (A2) holds true. For the second-order stationary process $\{X_{n}\}_{n\geq 1}$ with a common marginal distribution function, by (2.6), it follows that

3.19 $$ \begin{aligned}[b] \bigl\vert \sigma^{2}_{n}-n \sigma^{2}_{0} \bigr\vert &= \Biggl\vert n\gamma (0)+2n \sum_{j=1} ^{n-1} \biggl( 1-\frac{j}{n} \biggr) \gamma (j)-n\gamma (0) -2n\sum_{j=1}^{\infty } \gamma (j) \Biggr\vert \\ &= \Biggl\vert 2n\sum_{j=1} ^{n-1} \frac{j}{n}\gamma (j)+2n\sum_{j=n}^{\infty } \gamma (j) \Biggr\vert \leq 2\sum_{j=1}^{\infty }j \gamma (j)+2\sum_{j=n}^{ \infty }j\gamma (j) \\ &\leq 4\sum_{j=1}^{\infty }j \bigl\vert \gamma (j) \bigr\vert \leq 4C\sum_{j=1}^{\infty }j^{-\beta }=O(1). \end{aligned} $$

On the other hand,

3.20 $$ \begin{aligned}[b] &\sup_{-\infty < t< \infty } \biggl\vert P \biggl( \frac{\sum_{i=1}^{n}X _{i}}{\sqrt{n}\sigma_{0}}\leq t \biggr) -\Phi (t) \biggr\vert \\ &\quad \leq \sup_{-\infty < t< \infty } \biggl\vert P \biggl( \frac{\sum_{i=1}^{n}X _{i}}{\sigma_{n}} \leq \frac{\sqrt{n}\sigma_{0}}{\sigma_{n}}t \biggr) - \Phi \biggl( \frac{\sqrt{n}\sigma_{0}}{\sigma_{n}}t \biggr) \biggr\vert \\ &\qquad {}+ \sup_{-\infty < t< \infty } \biggl\vert \Phi \biggl( \frac{\sqrt{n}\sigma_{0}}{ \sigma_{n}}t \biggr) -\Phi (t) \biggr\vert \\ &\quad :=I_{1}+I_{2}. \end{aligned} $$

By (3.19), it is easy to see that $\lim_{n\rightarrow \infty }\frac{ \sigma_{n}^{2}}{n\sigma^{2}_{0}}=1$. Thus, applying Theorem 2.1, one has

3.21 $$ I_{1}\leq C \bigl\{ \bigl(\gamma_{1n}^{1/2}+ \gamma_{2n}^{1/2}\bigr)\log n+\gamma _{2n}^{(r-2)/2}+u^{1/3}(q) \bigr\} , $$

and according to (3.19) again, similar to the proof of (3.16), we obtain that

3.22 $$ I_{2}\leq C \biggl\vert \frac{\sigma_{n}^{2}}{n\sigma_{0}^{2}}-1 \biggr\vert = \frac{C}{n \sigma_{0}^{2}} \bigl\vert \sigma_{n}^{2}-n \sigma_{0}^{2} \bigr\vert =O\bigl(n^{-1}\bigr). $$

Combining (3.20), (3.21) with (3.22), (2.8) holds true. □

### Proof of Theorem 2.3

By taking the same notation as that in the proof of Theorem 1.1 in Yang et al. [1], denote $A=\sigma (\xi_{p})/f( \xi_{p})$ and

$$ G_{n}(t)=P \bigl( n^{1/2}(\xi_{p,n}- \xi_{p})/A\leq t \bigr). $$

Similar to the proof of (3.7) in Yang et al. [1], for $\beta >1$, we obtain that

$$ \bigl\vert \sigma^{2}(n,t)-\sigma^{2}(\xi_{p}) \bigr\vert =O \bigl( n^{-3/10}(\log n\log \log n)^{1/2} \bigr) +o \bigl(n^{-1/5}\bigr). $$

On the other hand, seeing the proof of (3.9) in Yang et al. [1], by Theorem 2.2, it follows that

$$ \begin{aligned}[b] &\sup_{ \vert t \vert \leq L_{n}} \bigl\vert G_{n}(t)-\Phi (t) \bigr\vert \\ &\quad \leq \sup_{ \vert t \vert \leq L_{n}} \biggl\vert P \biggl[ \frac{\sum_{i=1}^{n}Z_{i}}{\sqrt{n}\sigma (n,t)}< -c _{nt} \biggr] -\Phi (-c_{nt}) \biggr\vert +\sup_{ \vert t \vert \leq L_{n}} \bigl\vert \Phi (t)- \Phi (c_{nt}) \bigr\vert \\ &\quad \leq C \bigl\{ \bigl(\gamma_{1n}^{1/2}+\gamma_{2n}^{1/2} \bigr) \log n+\gamma_{2n}^{(r-2)/2}+u^{1/3}(q) \bigr\} + \sup_{ \vert t \vert \leq L_{n}} \bigl\vert \Phi (t)-\Phi (c_{nt}) \bigr\vert \\ &\quad \leq C \bigl\{ \bigl(\gamma_{1n}^{1/2}+\gamma _{2n}^{1/2}\bigr)\log n+\gamma_{2n}^{(r-2)/2}+n^{-1}+u^{1/3}(q) \bigr\} . \end{aligned} $$

Therefore, (2.9) follows the same steps as those in the proof of Theorem 1.1 of Yang et al. [1]. □

### Proof of Corollary 2.3

We obtain it by choosing $p=\lfloor n ^{\delta }\rfloor$, $q=\lfloor n^{2\delta -1}\rfloor $ in Theorem 2.3. □

## Appendix

### Lemma A.1

(cf. Yang [16])

*Let* *X* *and* *Y* *be random variables*, *then for any* $a>0$,

$$ \sup_{t} \bigl\vert P(X+Y\leq t)-\Phi (t) \bigr\vert \leq \sup_{t} \bigl\vert P(X\leq t)- \Phi (t) \bigr\vert + \frac{a}{\sqrt{2\pi }}+P\bigl( \vert Y \vert >a\bigr). $$

### Lemma A.2

(cf. Li [8])

*Let* $\{X_{j}\}_{j\geq 1}$ *be a LNQD random variable sequence with zero mean and finite second moment*, $\sup_{j \geq 1}EX^{2}_{j}<\infty $. *Assume that* $\{a_{j}\}_{j\geq 1}$ *is a real constant sequence*, $a:=\sup_{j}|a_{j}|<\infty $. *Then*, *for any* $r>1$, *there is a constant C not depending on* *n* *such that*

$$ E \Biggl\vert \sum_{j=1}^{n}a_{j}X_{j} \Biggr\vert ^{r}\leq Ca^{r}n^{r/2}. $$

### Lemma A.3

(cf. Wang [7])

*Let* $\{X_{n}\}_{n\geq 1}$ *be a sequence of LNQD random variables with* $EX_{n}=0$, $|X_{n}|\leq d$, *a*.*s*. *for* $n=1,2,\ldots $ . *Denote* $\vartriangle_{n}=\sum_{i=1}^{n}EX_{i} ^{2}$. *Then*, *for* $\varepsilon >0$ *and* $n\geq 1$,

$$ P \Biggl( \Biggl\vert \sum_{i=1}^{n}X_{i} \Biggr\vert >\varepsilon \Biggr) \leq 2 \exp \biggl\{ -\frac{\varepsilon^{2}}{2(2\vartriangle_{n}+d\varepsilon )} \biggr\} . $$

### Lemma A.4

(cf. Li [8])

*If* $X_{1},\ldots, X_{m}$ *are LNQD random variables with finite second moments*, *let* $\varphi_{j}(t_{j})$ *and* $\varphi (t_{1},\ldots, t_{m})$ *be c*.*f*.*’s of* $X_{j}$ *and* $(X_{1}, \ldots, X_{m})$, *respectively*, *then for all nonnegative*(*or non positive*) *real numbers* $t_{1},\ldots, t_{m}$,

$$ \Biggl\vert \varphi (t_{1},\ldots, t_{m})-\prod _{j=1}^{m}\varphi _{j}(t_{j}) \Biggr\vert \leq 4\sum_{1\leq l< k\leq m} \vert t_{l}t_{k} \vert \bigl\vert \operatorname{Cov}(X_{l},X_{k}) \bigr\vert . $$
